# Atherosclerosis Predictor? Circulating Levels of POPs Linked to Arterial Effects

**DOI:** 10.1289/ehp.120-a34a

**Published:** 2012-01-01

**Authors:** Julia R. Barrett

**Affiliations:** Julia R. Barrett, MS, ELS, a Madison, WI–based science writer and editor, has written for *EHP* since 1996. She is a member of the National Association of Science Writers and the Board of Editors in the Life Sciences.

Persistent organic pollutants (POPs) are long-lived environmental contaminants that are widely detected in humans. Elevated circulating levels of POPs have been linked to hypertension, obesity, diabetes, metabolic syndrome, and myocardial infarction in humans. Atherosclerosis, characterized by lipid-rich plaques on the inner wall (intima) of the artery, also can lead to myocardial infarction and stroke. A new study now shows an association between increased circulating levels of POPs and markers of atherosclerosis in the carotid artery [*EHP* 120(1):38–43; Lind et al.].

Atherosclerosis begins with low-density lipoprotein cholesterol accumulating beneath the intima. As the cholesterol undergoes oxidation, chronic inflammation leads to long-term arterial damage. Atherosclerosis is often accompanied by a thickening of the intima and the muscular layer, or media, of the artery. Increased intima-media thickness (IMT) and decreased echogenicity, or density, of the intima-media complex also predict future disease.

The current study drew on data from the Prospective Investigation of the Vasculature in Uppsala Seniors, a Swedish study involving 1,016 70-year-old men and women. Twenty-three POPs were measured in the participants’ blood, including 16 polychlorinated biphenyl (PCB) congeners, 5 pesticides, 1 dioxin, and 1 brominated compound. Twenty-one POPs were found in at least 90% of the participants.

Participants underwent ultrasound examination of their carotid arteries for plaque detection and IMT measurement. Ultrasound images were also used in gray-scale computer analysis to determine the echogenicity of the intima-media complex. Several individual PCB congeners as well as the sum of PCB congeners were associated with plaque presence, and highly chlorinated PCBs were inversely associated with echogenicity of the intima-media complex. The sum of the toxic equivalents of the PCBs and the dioxin also was inversely associated with echogenicity and positively associated with IMT. The relationships remained significant even after controlling for multiple confounding factors, including known cardiovascular risk factors.

This study builds upon earlier findings of an association between increased circulating levels of POPs and myocardial infarction. The results also suggest that POPs have vascular effects that are independent of known risk factors for atherosclerosis, potentially affecting the development of atherosclerosis at several points, e.g., through altered expression of genes related to inflammation. Given the restricted study population, the findings cannot be generalized to more ethnically diverse groups of different ages. Prospective studies are needed to examine the suggested effects of POPs on myocardial infarction risk.

